# Utility of registries for post-marketing evaluation of medicines. A survey of Swedish health care quality registries from a regulatory perspective

**DOI:** 10.1080/03009734.2017.1285837

**Published:** 2017-03-03

**Authors:** Nils Feltelius, Rolf Gedeborg, Lennart Holm, Björn Zethelius

**Affiliations:** Swedish Medical Products Agency, Uppsala, Sweden

**Keywords:** Drug regulation, health technology assessment, medical devices, pharmacoepidemiology, quality registries, real world data

## Abstract

**Aim:**

The aim of this study was to describe content and procedures in some selected Swedish health care quality registries (QRs) of relevance to regulatory decision-making.

**Methods:**

A workshop was organized with participation of seven Swedish QRs which subsequently answered a questionnaire regarding registry content on drug treatments and outcomes. Patient populations, coverage, data handling and quality control, as well as legal and ethical aspects are presented. Scientific publications from the QRs are used as a complementary measure of quality and scientific relevance.

**Results:**

The registries under study collect clinical data of high relevance to regulatory and health technology agencies. Five out of seven registries provide information on the drug of interest. When applying external quality criteria, we found a high degree of fulfillment, although information on medication was not sufficient to answer all questions of regulatory interest. A notable strength is the option for linkage to the Prescribed Drug Registry and to information on education and socioeconomic status. Data on drugs used during hospitalization were also collected to some extent. Outcome measures collected resemble those used in relevant clinical trials. All registries collected patient-reported outcome measures. The number of publications from the registries was substantial, with studies of appropriate design, including randomized registry trials.

**Conclusions:**

Quality registries may provide a valuable source of post-marketing data on drug effectiveness, safety, and cost-effectiveness. Closer collaboration between registries and regulators to improve quality and usefulness of registry data could benefit both regulatory utility and value for health care providers.

## Introduction

In Europe, assessment of the safety and efficacy of a new drug before approval and during its entire life-cycle is performed within the European network of regulatory agencies, in Sweden represented by the Medical Products Agency (*Läkemedelsverket*). This regulatory collaboration is coordinated by the European Medicines Agency (EMA). Decisions made within this regulatory network have important implications for the availability of safe drugs and vaccines to safeguard public health. In 2015 EMA set up a so-called Cross-Committee Registry Task Force to promote the use of data from disease registries for regulatory purposes. As a contribution to this effort the Medical Products Agency (MPA) in collaboration with national quality of care registries (QRs) performed a survey to describe the utility of registry-based clinical data generation in Sweden. Registries delivering high-quality data on drug exposure and/or relevant outcomes in clinical practice are valuable assets in the assessment of drug safety and effectiveness for all stakeholders. At present the knowledge of how far registries actually can meet this need is limited. This inventory of Swedish quality registries aims at filling this knowledge gap.

In Sweden different types of registries containing health care data have been organized to support clinical decision-making, quality improvement, as well as health technology assessment and policy-making. At the national level there are governmental National Health Care Registries (NHCR) held by the National Board of Health and Welfare (e.g. the Patient Registry, Cancer Registry, Cause of Death Registry, Prescribed Drug Registry [PDR], and Birth Registry) covering the entire Swedish population and with mandatory reporting. Regional health care databases cover county and regional populations, and there are also the QRs—the focus of this survey—that provide nationwide data, usually encompassing a specific disease, intervention, or patient group. QRs have been set up at the initiative of health care professionals primarily to support the improvement and sustainability of quality of care. In Sweden there are more than one hundred QRs, but the vast majority do not collect data on drug treatment (1). Data from QRs can be linked—by use of the personal identification number (PIN) given to all permanent residents in Sweden (2)—to other registries. In contrast to product registries which collect information on a single drug product, most QRs cover a disease which allows comparative studies. The utility of linkage to the Prescribed Drug Registry is reflected by the substantial output in the scientific literature as recently reviewed by Wallerstedt et al. (2).

International collaborations between registries, including Swedish QRs, have provided useful data (3,4). However, pooling individual-level data from registries located in different countries often raises problems of legal as well as logistic nature that may necessitate specific considerations (5,6). If such problems are overcome, these studies may enable comparison between country-specific settings or increase the size of patient populations for studies of orphan diseases or other situations with rare outcomes (7).

To describe the potential of QRs to meet a growing regulatory need for data from clinical practice we performed a survey including a selected subset of registries. All of these represent therapeutic areas of importance to public health and where new drugs have recently been introduced with requirements for post-marketing follow-up.

## Methods

### Definition of a national quality of care registry

The majority of Swedish QRs are organized and run by the medical profession. The aim is to improve the care of patients with a specific disease or the quality and outcome of a certain medical intervention. The registry is integrated in daily practices and sometimes also supports clinical decision-making. It is financed by public funding and governed by national laws and regulations (8).

### Selection of registries

To be included, the registry should fulfill the national requirements for certification level 1 or 2 according to the Swedish Association of Local Authorities and Regions (SALAR). Requirements for certification level 1 include direct information to patients on registry results, active use of data for research and obtaining research funding in national or international competition, systematic validation of data quality, and control of coverage by cross-checking versus other data sources. The following five registries are certified at level 1: NDR (National Diabetes Registry), SRQ (Swedish Rheumatology Quality Registry), SWEDEHEART (The Swedish Web-system for Enhancement and Development of Evidence-based care in Heart disease Evaluated According to Recommended Therapies), Riksstroke, and NPCR (National Prostate Cancer Registry of Sweden); and the following two at level 2: SMSreg (Swedish Multiple Sclerosis Registry) and MACULAREG (Macula Registry). All registries are known to systematically collect data on drug treatment, and report at least five peer-reviewed publications in the field of drug efficacy or safety, facts also deciding selection for the study. These registries represented cardiology, neurology, multiple sclerosis (MS), stroke, rheumatoid arthritis, diabetes, prostate and breast cancer, and ophthalmology. All participated in a workshop organized by the MPA where the purpose of the project was presented and acceptance to participation confirmed. One invited registry, the breast cancer registry, chose not to participate.

### Collection of information

The questionnaire used in this survey has been used previously (5,9). The questionnaire (MPA Quality Registry Questionnaire, All Rights Reserved; available online) was used to extract basic administrative data and information on data collection, handling, quality assurance, reporting, ethical and legal aspects, funding, and governance. Although the questionnaire was not formally validated, the reliability of data was controlled as registry holders verified on two occasions that the information from their respective registry was correctly transferred from the questionnaire to the tabular presentation in this report.

### Assessment of regulatory usefulness

The information in the questionnaires was used to describe the usefulness of the QRs with focus on the following aspects: Completeness (number of participating units), Coverage (proportion of eligible patients included), Validity (clinically relevant and quality-assured data), Comparability (i.e. definitions and outcomes identical to those used in randomized controlled trials [RCTs], possibility to create control groups), and Organizational and financial robustness. The usefulness of QR data was assessed also from the regulatory relevance of their scientific publications dealing with drug-related issues. To illustrate this further, the publications were subdivided and presented in categories of safety, effectiveness, health economics, and issues on methodology, the last-mentioned category also including aspects on multinational collaboration.

To apply an external perspective to our description we used two sets of criteria elaborated by the National Institute for Health and Care Excellence (NICE) (10,11).

The first set is suggested to improve the quality of evidence generation for new treatments (i.e. when setting up a registry) and points out five areas of particular importance (10). These are: 1) Establishing a management structure; 2) Agreeing a mandatory data subset; 3) Preventing and monitoring incoherent entries; 4) Motivating those submitting data; and 5) Triangulation and data linkage of registry data to external data sources.

The second NICE set proposes the use of six main criteria when assessing the quality of a registry, which can be summarized as follows (11): 1) Data completeness in terms of patient population (as denominator); 2) Relevance of the data for answering the question; 3) Data granularity; 4) Independence of the registry; 5) Publications with data made from the registry; and 6) Aspects of data protection.

## Results

### Organizational aspects

Some QRs have formed an umbrella organization containing several subregistries (Table 1). This has permitted new subregistries to develop from an ancestral QR (e.g. neuro-registry from MS only to Parkinson’s disease, myasthenia, narcolepsy, etc.) which has provided IT platforms and practical experience facilitating the inclusion of additional diagnoses. In other cases QRs has evolved in the other direction, i.e. there has been a merger of initially independent registries into one larger body (e.g. SWEDEHEART). Some of the QRs have been active for 15 years or more, which has allowed the development of high coverage and robust systems for collaboration. All but one registry have websites in English facilitating contact with external parties like regulators, drug companies, academic groups, and other international stakeholders.

**Table 1.** Administrative information on eight Swedish Health Care Quality Registries included in the survey.

Registry (abbreviation, certification level)^a^	Target patient population	Subregistries, subprojects	Website	Website in English
Swedish Multiple Sclerosis Registry (SMSreg, 2)	Multiple sclerosis	Under the heading of NEUROreg, there are 7 subregistries besides SMSreg: Parkinson's disease, narcolepsy, myasthenia gravis, inflammatory polyneuropathy, epilepsy, severe vascular headache, motor neuron disease	www.neuroreg.se	http://www.neuroreg.se/en.html
National Prostate Cancer Registry of Sweden (NPCR, 1)	Prostate cancer	Five-year follow-up study	www.npcr.se	www.npcr.se/in English
Riksstroke (Riksstroke, 1)	Stroke and TIA	Childhood stroke module launched 1 January 2016	www.riksstroke.org/swe/	http://www.riksstroke.org/eng/
Swedish Macula Registry (MACULAREG, 2)	Diseases of the macula of the eye and associated complications, age-related wet macular degeneration, myopia, chronic retinal central serosa, inflammation, angioid streaks, trauma, idiopathic, macular telangiectasia, other.	Thrombosis registry (CRVO = central retinal vein occlusion; BRVO = branch retinal vein occlusion; HRVO = hemi retinal vein occlusion)	http://rcsyd.se/sm/	Not established in English
Swedish National Diabetes Registry (NDR, 1)	Diabetes mellitus (types 1 and 2)	SWEDIABKIDS (<18 y)	www.ndr.nu	https://www.ndr.nu/#/english
Swedish Rheumatology Quality Registry (SRQ, 1)	Rheumatoid arthritis, psoriatic arthritis, ankylosing spondylitis	Myositis	www.srq.nu	http://srq.nu/en/
SWEDEHEART—The Swedish Web-system for Enhancement and Development of Evidence-based care in Heart disease Evaluated According to Recommended Therapies (SWEDEHEART, 1)	Acute coronary syndromes, myocardial infarction, coronary angiography, percutaneous coronary intervention, coronary by-pass surgery, cardiothoracic surgery, percutaneous valve intervention (TAVI/mitral), cardiogenetic disorder	RIKS-HIA: Coronary care, acute coronary syndromes, myocardial infarction; SEPHIA: Secondary prevention after myocardial infarction; SCAAR: Coronary angiography, percutaneous coronary intervention; Swedish heart surgery registry (HKIR): Cardiothoracic surgery, coronary by-pass surgery; Percutaneous Valve Registry (PVR): percutaneous valve intervention (TAVI/mitral), cardiogenetic disorders	http://www.ucr.uu.se/swedeheart/	http://www.ucr.uu.se/swedeheart/index.php/dokument-sh/arsrapporter Information on SWEDEHEART is available in: Jernberg T Heart. 2010 Oct;96(20):1617-21 (PMID: 20801780), Lagerqvist B Engl J Med. 2014 Sep 18;371(12):1111-20/web appendix (PMID: 25176395)

a Certification level is a rating given to each registry and represents the level of development the registry has reached in terms of analyses, inclusion of relevant indicators, coordination with health services, use in research, data quality and reporting, coverage rate, technical solutions/tools, etc. There are four levels in total: 1 (highest), 2, 3, and the candidate level (lowest) (http://kvalitetsregister.se/englishpages/findaregistry/certificationlevels.2029.html).

### Patient selection and coverage

The number of included patients varied from 21,439 in the ophthalmological registry to more than 1.5 million in the cardiology registry (Table 2). Coverage of the target population was high, for all estimated to be above 80%. It should be noted that registries classified as ‘interventional’ have coverage of 100%.

**Table 2.** Information on inclusions and coverage of quality of care registries (autumn 2015).

Registry		SMSreg	NPCR	Riksstroke	MACULAREG	NDR	SRQ	SWEDEHEART
2. 1	Total cumulative no. of patients included:	16,800	150,000	440,000	21,439	500,000	64,947	>1.5 million
2. 2	No. of patients included per year (approx. mean last 3 years):	2,800	10,000	24,000	3,700	384,124 (no. of updates per 2015)	9,800	80,000 **Subregistries**^a^: RIKS-HIA: 44,089 care episodes SEPHIA: 60,000 patients since 2005, follow-up data for 6,532 patients SCAAR: 39,965 coronary angiographies HKIR: Number of registered operations/procedures =5,613 PVR: 425 TAVI procedures
2. 3	No. of participating centers/clinics out of eligible units:	60	54	72	38	1,260 (= health care centers); 90 (= hospitals)	60	73
2. 4	No. of eligible centers/clinics:^b^	60	54	72	41^c^	–	60	73
2. 5	Proportion of eligible patients (=coverage) included in the registry (%):	83	98	91	80	95	82	100% for the interventional registries, >90% of all cases of myocardial infarction
2.6	How is coverage calculated (defined by each registry)?	Patients in MSreg compared to national prevalence	Compared to Cancer Registry	Proportion of patients with 1st stroke in Riksstroke compared to patients with 1st stroke diagnosis in Patient Registry	Comparison with PAR (national Patient Registry)	Comparison between the NDR and the nationwide Prescribed Drug Registry	Comparison of data from SRQ and national Patient Registry	Cases in registry versus cases in public mandatory registries (PAR)

a Data from 2014; source: http://www.ucr.uu.se/swedeheart/index.php/dokument-sh/arsrapporter/doc_download/392-swedeheart-arsrapport-2014-english-engelsk.

b Hospital clinics, hospital outpatient clinics, primary health care centers.

c Data from 2014 annual report.

The majority of the QRs can recruit patients and controls for clinical studies and also have the option to randomize to treatment within the registry (randomized registry controlled trials, RRCTs) (Table 3).

**Table 3.** Patient population and controls.

3.	Data content/elements:	Yes (*n*)	No (*n*)	Yes—which registry	No—which registry
3. 1	Are patients participating in ongoing RCTs included in the registry? (Y/N)	4	3	SMSreg, NPCR, SRQ, SWEDEHEART	Riksstroke, MACULAREG, NDR
3. 2	Are RRCTs possible to perform within your registry? (Y/N)	6	1	SMSreg, NPCR, Riksstroke, MACULAREG, SRQ, SWEDEHEART	NDR: in future, yes
3. 3	Is it possible to create a control group? (Y/N)	6	1	NPCR, Riksstroke, MACULAREG, NDR, SRQ, SWEDEHEART	SMSreg
3. 4	Are patients from other countries included in the registry? (Y/N)	2	5	SWEDEHEART: ‘Iceland directly, Norway in a parallel SCAAR registry’, NDR: ‘Iceland—SWEDIABKIDS’	SMSreg, NPCR, Riksstroke, MACULAREG, SRQ
3. 5	Demographic limitations, e.g. age group, geographical? (Y/N)	1	6	NDR	SMSreg, NPCR, MACULAREG, Riksstroke, SRQ, SWEDEHEART
	If Yes, please specify			>18 y^a^	SWEDEHEART–SEPHIA: Age limit of <75 years

a In NDR patients, >18 y is registered; if <18 y, SWEDIABKIDS is used.

### Data recorded

Data recorded in the QRs are—for natural reasons—to a large extent disease-specific (Table 4). For registries focusing on interventional procedures the principal diagnosis may vary, as the inclusion is decided by the intervention and not the disease. Information on patients (sex, age, etc.) and on the disease in question (duration, scores for disease activity and organ damage), physical function, patient-reported outcome measures (PROMs), etc. is provided by all QRs or can be retrieved by linkage. Information on education and socioeconomic status can be obtained by linkage to other national registries, held by Statistics Sweden. The information on medication is of varying quality. Five out of seven registries provide information on the drug of interest, i.e. a targeted follow-up is included in the data collection. All prescribed medications can be found in the Prescribed Drug Registry and linked to other data by the PIN. Data on drugs used for inpatient care (i.e. non-prescribed) are collected by the QRs included in this survey. The outcome measures collected in the QRs are to a high extent the same as those used in the relevant clinical trials. Long-term safety can be adequately followed by means of data collected within each QR but importantly through the PIN and the possibility for linkage to other data sources. Some of the QRs are connected to the MPA for direct electronic reporting of suspected adverse drug reactions (ADRs).

**Table 4.** Data recorded in the Quality of Care Registries.

		Yes (*n*)	No (*n*)	Yes—which registry	No—which registry
1. Data elements registered at inclusion in registry					
1. 1	Age (Y/N)	7	0	All	
1. 2	BMI (Y/N)	2	5	NDR, SWEDEHEART	SMSreg, NPCR, Riksstroke, MACULAREG, SRQ
1. 3	Sex (Y/N)	7	0	All	
1. 4	Diagnosis (Y/N)	7	0	All	
1. 5	Comorbidities (Y/N)	4	3	Riksstroke, NDR, SRQ, SWEDEHEART	SMSreg, NPCR, MACULAREG
1. 6	What terminology for coding of diagnosis and comorbidity is used?				
	ICD-10	6	1	SMSreg, NPCR, Riksstroke, NDR, SRQ, SWEDEHEART	MACULAREG
	Other	2	5	MACULAREG, SWEDEHEART	SMSreg, NPCR, Riksstroke, NDR, SRQ
	Free text	0	7		All
1. 7	Time point for disease onset (Y/N)	7	0	All	
1. 8	Disease activity/state (Y/N)	7	0	All	
	If Yes, please specify used measurement	Riksstroke: Level of consciousness, functional dependence at onset; functioning at 3 and 12 months; SRQ: DAS28, HAQ, BASDAI, EQ-5D; SWEDEHEART: Shock, Killip class, severity and distribution, duration of symptoms			
2. Medication—drug of interest						
2. 1	Is indication for treatment with drug of interest recorded in the registry? (Y/N)	7	0	All	
2. 2	If yes, what terminology for coding of indication is used?	–	–	–	–
2. 3	What elements concerning medication are recorded?				
	Product (Y/N)	5	2	SMSreg, NPCR, MACULAREG, NDR, SRQ	Riksstroke, SWEDEHEART
	Substance (Y/N)	6	1	SMSreg, NPCR, Riksstroke, NDR, SRQ, SWEDEHEART	MACULAREG
	ATC code (Y/N)	2	5	NDR, SRQ	SMSreg, NPCR, Riksstroke, MACULAREG, SWEDEHEART
	Dosage (Y/N)	3	4	SMSreg, NDR, SRQ	NPCR, Riksstroke, MACULAREG, SWEDEHEART
	Duration/exposure (Y/N)	4	3	SMSreg, MACULAREG, NDR, SRQ	NPCR, Riksstroke, SWEDEHEART
	Therapy start/stop date (Y/N)	5	2	SMSreg, Riksstroke, MACULAREG, NDR, SRQ	NPCR, SWEDEHEART
	Is reason for stop/switch to other drug registered? (Y/N)	3	4	SMSreg, NDR, SRQ	NPCR, Riksstroke, MACULAREG, SWEDEHEART
2. 4	Is concomitant medication recorded in your registry? (Y/N)	2	5	SRQ: ‘concerning rheumatic disease’, SWEDEHEART	SMSreg, NPCR, Riksstroke, MACULAREG, NDR: Data have been linked to Prescribed Drug Registry for recognition of concomitant drugs
3. Information regarding follow-up					
3. 1	Are follow-up visits recorded in your registry? (Y/N)	6	1	SMSreg, MACULAREG, NDR, SRQ, SWEDEHEART, Riksstroke	NPCR
3. 2	Are follow-up visits scheduled at regular intervals? (Y/N)	4	3	MACULAREG, NDR, SRQ: ‘when a new drug is started’, SWEDEHEART	SMSreg, NPCR, Riksstroke
	If Yes, please specify intervals				
	Regularly	2	5	NDR, SWEDEHEART	SMSreg, NPCR, Riksstroke MACULAREG, SRQ
	Ad hoc	0	7		All
	Both	3	4	SMSreg, MACULAREG, SRQ	NPCR, Riksstroke, NDR, SWEDEHEART
3. 3	Are patients lost to follow-up registered?^a^ (Y/N)	3	4	SMSreg, Riksstroke, SRQ	NPCR, MACULAREG, NDR, SWEDEHEART: ‘No lost to follow-up based on public registry data. For SEPHIA visits there may be lost to FU’
3. 4	Are reasons for loss to follow-up registered? (Y/N)	1	6	SRQ	SMSreg, NPCR, Riksstroke, MACULAREG, NDR, SWEDEHEART
3. 5	Maximum duration of long-term follow-up?			SMSreg: ‘no limit’; NPCR: 16 y; Riksstroke: 1 y; MACULAREG: 15 y; NDR: 20 y; SRQ: ‘approx. 24 months but increasing length’; SWEDEHEART: ‘No lost to follow-up based on public registry data’	
3. 6	Is ongoing medication with drug of interest registered at follow-up? (Y/N)	5	2	SMSreg, MACULAREG, NDR SRQ, SWEDEHEART: In SEPHIA	NPCR, Riksstroke
3. 7	Is it possible to follow-up teratogenic events, due to medication with drug of interest? (Y/N)	0	6		SMSreg, NPCR, Riksstroke, MACULAREG, SRQ, SWEDEHEART
3. 8	Is bio-banking of DNA or tissue samples performed at inclusion? (Y/N)	2	5	SMSreg, SWEDEHEART: ‘For patients with MI at selected sites’	NPCR, Riksstroke, MACULAREG, NDR SRQ
3. 9	Is bio-banking of DNA or tissue samples performed at follow-up? (Y/N)	2	5	SMSreg, SRQ	NPCR, Riksstroke, MACULAREG, NDR, SWEDEHEART
4. Outcome measures					
4. 1	Disease activity/state (Y/N)	5	2	SMSreg, Riksstroke, NDR, SRQ, SWEDEHEART	NPCR, MACULAREG
4. 2	Organ damage, e.g. renal damage (Y/N)	3	4	NDR, SRQ, SWEDEHEART	SMSreg, NPCR, Riksstroke, MACULAREG
4. 3	Physical function (Y/N)	6	1	SMSreg, Riksstroke, MACULAREG, SRQ, SWEDEHEART, NDR: ‘physical activity’	NPCR
4. 4	Health economy/cost-effectiveness data (Y/N)	4	3	Riksstroke, NDR, SRQ, SWEDEHEART	SMSreg, NPCR, MACULAREG
4. 5	PROM (patient-reported outcome measure) (Y/N)	7	0	All (SWEDEHEART: ‘Recently started at selected sites’)	
4. 6	PREM (patient-reported experiences measure) (Y/N)	4	3	SMSreg, NPCR, Riksstroke, MACULAREG	NDR, SRQ, SWEDEHEART
4. 7	Is information on deaths and cause of death recorded? (Y/N) (MPA comment: All registries can link data to Cause of Death Registry, after ethical approval)	5	2	SMSreg, Riksstroke: ‘Not cause of death’, NDR, SRQ: ‘Only information on death’, SWEDEHEART	NPCR, MACULAREG
4. 8	Quality of life (Y/N)	6	1	SMSreg, NPCR, SRQ, SWEDEHEART: ‘In SEPHIA and percutaneous valves’, NDR, Riksstroke: ‘general health condition’	MACULAREG
4. 9	Can you provide a summary list of used outcome measures in English?	6	1	SMSreg, NPCR, SRQ, SWEDEHEART, NDR: on demand, Riksstroke	MACULAREG
4. 10	Are the outcome measures the same as those used in clinical trials?	7	0	All	
4. 11	Can long-term safety be followed within your registry? (Y/N)	7	0	All (SWEDEHEART: ‘Yes and no—for selected variables. Stent thrombosis and restenosis in SCAAR’)	
4. 12	If yes, is linkage to other data sources required? (Y/N)	6	1	SMSreg, NPCR, MACULAREG, NDR, SRQ, SWEDEHEART: ‘Yes and no—depends on safety variables’	Riksstroke
5. Adverse event detection, processing, and reporting					
5. 1	Are adverse drug reactions (ADRs) registered within registry? (Y/N)	5	2	SMSreg, MACULAREG, SRQ, SWEDEHEART: ‘Contrast media and hemodynamic reactions noted in SCAAR’, Riksstroke: intracerebral hemorrhage during anticoagulant therapy, and from thrombolytic therapy are recorded	NPCR, NDR
	If Yes, is an approved terminology for coding of ADRs used, i.e. MedDRA? (Y/N)	2	5	SMSreg, SRQ	NPCR, Riksstroke, MACULAREG, NDR, SWEDEHEART
	Does the registry provide means for web-based reporting of ADRs directly to MPA?	2	5	SMSreg, SRQ	NPCR, Riksstroke, MACULAREG, NDR, SWEDEHEART
5. 2	Are events (not ADRs) interfering with medication (surgery, accidents, etc.) registered? (Y/N)	1	6	NDR: bariatric surgery	SMSreg, NPCR, Riksstroke, MACULAREG, SRQ, SWEDEHEART

^a^MPA comment: Migration and death of registered patients can be traced by registry linkage in all registries.

### Quality control procedures

The majority of the QRs have well-defined quality control procedures in place (Table 5). If specific research studies are performed using registry data, ethics approval and patient consent are obtained according to standard requirements and applicable legislation. As the collection of data for improvement of health care quality is seen as a part of routine care, specific permissions are not necessary. The basic regulation of this is laid down in the Swedish Personal Data Act (12) and the specific Patient Data Act (13), resulting in uniform processing of patient data by all registries.

**Table 5.** Data management and quality control.

		Yes (*n*)	No (*n*)	Yes—which registry	No—which registry
1. Data capture/entry into database by:					
1. 1	Web-based reporting into database? (Y/N)	7	0	All	
1. 2	Is it possible for patients to enter PROMs directly into the registry by the web? (Y/N)	3	4	SMSreg, SRQ, SWEDEHEART	NPCR, Riksstroke: ‘planned for 2016’, MACULAREG, NDR: ‘Ongoing’
1. 3	Is data in the registry extracted directly from electronic health records? (Y/N)	3	4	NDR, SRQ, SWEDEHEART	SMSreg, NPCR, Riksstroke, MACULAREG
1. 4	If yes, percentage of participating health care centers with this opportunity?			NDR: 67%; SRQ: ‘From 1 EMR system’	
1. 5	Is the database an integrated part of an electronic patient record system? (Y/N)	1	6	NDR	SMSreg, NPCR, Riksstroke, MACULAREG, SRQ, SWEDEHEART
2. Quality control					
2. 1	Is there a specifically qualified person for quality control of data? (Y/N)	4	3	Riksstroke, SRQ, SWEDEHEART, NDR	SMSreg, NPCR, MACULAREG
	If yes, please specify qualification (MD, research nurse, inspector, etc.)	SMSreg: ‘Nurses … at the end of 2015’; Riksstroke: ‘Statistician’; SRQ: ‘… network of quality persons, MDs, nurses’; SWEDEHEART: ‘7 regional monitors are monitoring all sites’; NDR: ‘regional quality coordinators’			
2. 2	Do you apply recommendations from specific international quality guidelines? (Y/N)	4	3	SMSreg, NPCR, Riksstroke, NDR	MACULAREG, SRQ, SWEDEHEART
	If yes, please specify	SMSreg: ‘… recommendations from the Swedish MS Society … based on international guidelines’; NPCR: ‘Swedish Guidelines’; Riksstroke: ‘Recommendations/guidelines from Swedish national board of health and welfare and the European Stroke Organization’; NDR: ‘ADA/EASD guideline recommendations on diabetes care are in principal adopted as these are reflected in applicable Swedish national GLs, published by the National Board of Health and Welfare, to be used in diabetes care and also treatment recommendations on use of medicinal products within the field are published by the Medical Products Agency. The latter is under revision aiming to be published in 2017’			
2. 3	At what frequency is quality check performed (of raw data, delivery, etc.)?				
	Regularly	4	3	Riksstroke, SRQ, SWEDEHEART, NDR: yearly	SMSreg, NPCR, MACULAREG
	Randomly	2	5	SMSreg, SRQ	NPCR, Riksstroke, MACULAREG, SWEDEHEART, NDR
	Event-driven	3	4	NPCR, MACULAREG, SRQ	SMSreg, NDR, Riksstroke, SWEDEHEART
2. 4	At what level is registry data stored?				
	Local/regional	1	6	SRQ, NDR	SMSreg, NPCR, Riksstroke, MACULAREG, SWEDEHEART
	National	7	0	All	
	Multinational	0	7		All
2. 5	Are missing data actively requested? (Y/N)	5	2	NPCR, Riksstroke, NDR, SRQ, SWEDEHEART	SMSreg, MACULAREG
2. 6	Do you consider it possible to request additional information from treating physician, if needed by external stakeholders (e.g. pharma companies)? (Y/N)	2	5	NDR, SWEDEHEART	SMSreg, NPCR, Riksstroke: ‘Only with a new research application to ethics committee’, MACULAREG, SRQ

Ethics committee approval is sought for all scientific projects, including all linkage studies (Table 6).

**Table 6.** Ethical aspects.

		Yes (*n*)	No (*n*)	Yes—which registry	No—which registry
1. 1	Is there written patient information?	7	0	All	
1. 2	Is a formal patient consent obtained?	5	2	MACULAREG, NDR, SRQ, SWEDEHEART (see next row)	SMSreg, Riksstroke, NPCR
	If yes, how?				
	Written consent	2	5	NDR, SWEDEHEART: ‘written consent only for bio-bank’	SMSreg, NPCR Riksstroke, MACULAREG, SRQ
	Verbal consent	4	3	Riksstroke, MACULAREG, NDR, SRQ	SMSreg, NPCR, SWEDEHEART
1. 3	Does consent include an agreement to ask for follow-up information by e.g. a questionnaire, when needed from stakeholders (e.g. pharma companies)? (Y/N)	3	3	SMSreg, MACULAREG, SWEDEHEART	NPCR, Riksstroke, SRQ
1. 4	Has an ethics committee approved the working procedures/protocols of your registry? (Y/N)	5	2	SMSreg, Riksstroke, MACULAREG, NDR, SWEDEHEART	NPCR, SRQ
1. 5	Has your registry adopted any specific code of conduct, e.g. Helsinki declaration or ENCEPP’s code of conduct? (Y/N)	3	4	SMSreg, Riksstroke, NDR	NPCR, MACULAREG, SRQ, SWEDEHEART

### Governance

All registries are owned by public/governmental bodies (Table 7). The funding for running the QRs is public, and yearly applications are needed (8). Decisions regarding funding are made by a committee nominated by the Government.

**Table 7.** Legal, organizational, and financial aspects.

		Yes (*n*)	No (*n*)	Yes—which registry	No—which registry
1. Legal and organizational aspects					
1. 1	If available, please provide an organogram of your registry set-up	1	0	MACULAREG	
1. 2	Who owns the registry data?				
	County council^a^	7	0	All	
	Academic institution	0	7		All
	Pharma company	0	7		All
1. 3	Is there a formal (written) agreement between participating centers regulating data handling and analytic procedures? (Y/N)	2	5	SMSreg, NDR	NPCR, Riksstroke, MACULAREG, SRQ, SWEDEHEART
1. 4	Do you collaborate with pharma companies, based on data from the registry? (Y/N)	4	3	MACULAREG, NDR, SRQ, SWEDEHEART	SMSreg: ‘But academic units may research registry data being sponsored by pharma’, NPCR, Riksstroke
1. 5	If yes, are the results used by companies for regulatory purposes? (Y/N)	4	2	SMSreg, SRQ, SWEDEHEART	Riksstroke, MACULAREG
1. 6	Is patient privacy protected by specific measures? (Y/N)	5	2	NPCR, MACULAREG, NDR, SRQ, SWEDEHEART	SMSreg, Riksstroke
1. 7	If yes, how? Data/sample coding?	NPCR: ‘remote server data stripped of identifier’; MACULAREG: ‘coding’			
1. 8	Do you have a direct communication/exchange of information with national regulatory agency (MPA)? (Y/N)	6	1	NDR, SRQ, NPCR, SMSreg: ‘Adverse events reported go directly to MPA’, MACULAREG: ‘We are sharing data to compare systemic adverse events’, SWEDEHEART: ‘reporting on stent performance’	Riksstroke
2. Financial aspects					
2. 1	Funding by governmental/health care authorities? (Y/N)	7	0	All	
	Approx. proportions (%) of total sum from each contributing part?			SMSreg: 100%; NPCR: 90%; SRQ: 75%; Riksstroke: 100%	
2. 2	By industry? (Y/N)	1	6	SRQ	SMSreg, NPCR, Riksstroke, MACULAREG, NDR, SWEDEHEART
	Approx. proportions (%) of total sum from each contributing part?				SRQ: 25%	
2. 3	By research grants? (Y/N)	1	6	NPCR	SMSreg, Riksstroke, MACULAREG, NDR, SRQ, SWEDEHEART
	Approx. proportions (%) of total sum from each contributing part?	–	–		

a Regional public health authority is responsible in accordance with data protection regulations. Data collection, management, and reporting are led by a steering group nominated by the relevant health care professional organization.

The financial and organizational robustness of these QRs seem reassuring as the funding comes from public sources and the governance is firmly integrated in the clinical professional organizations and the County Councils. Details on the proportions of public versus other funding were not asked for in this survey.

### Reporting

Information on the results and specific studies is presented in scientific publications and in yearly reports to the Funding Committee and the County Councils and to the public. Some of the QRs provide feed-back to the participating physicians in real time through internet-based interactive reporting. The last-mentioned provides an important professional incentive to participate and efficiently counteracts ‘reporting fatigue’. The MS registry has the most elaborate real-time feed-back to reporting physicians (Table 8). For the within-registry communication, real-time feed-back of aggregated data at national, regional, and hospital level is becoming increasingly important. As the annual reports are key components in applications for continued public funding, they are comprehensive and give a good overview of the status of the registry. Registry data are also discussed at meetings with the respective national professional society. However, the scientific publications are the most important way to inform of results from the registries at the international level.

**Table 8.** Communication and reporting.

		Yes (*n*)	No (*n*)	Yes—which registry	No—which registry
8. 1	Do you communicate results from your registry by scientific publications? (Y/N)	7	0	All	
8. 2	If by other means, please specify, e.g. annual report	All registries communicate by an annual report			
8. 3	How many scientific publications have been published the last two years, based on data from your registry?	SMSreg: 51; NPCR: 30 plus; Riksstroke: 40; MACULAREG: 1; NDR: 35; SRQ: 85; SWEDEHEART: ‘Approx. 100’			
8. 4	When is feed-back given to reporting physician/clinics?				
	In real-time (Y/N)	7	0	All	
	Annually (Y/N)	7	0	All	
	Ad hoc, in case of need, e.g. safety problems (Y/N)	6	1	SMSreg, Riksstroke, MACULAREG, NDR, SRQ, SWEDEHEART	NPCR

### Applying external quality criteria to the registry content and procedures

When applying the NICE criteria to the registries, we found that all QRs had taken such aspects into consideration when setting up their registry as well as when performing quality control over time.

However, the item ‘granularity’, i.e. detailed information on medication, was not sufficient to answer all questions of regulatory interest.

### Registry of scientific publications of regulatory relevance

A selection of publications from the registries is presented in a Supplementary Table (Publications of Regulatory Significance; available online) to illustrate their potential regulatory significance. They cover a broad range of scientific issues including drug safety, effectiveness, and utilization relating to multiple sclerosis, cardiovascular diseases, rheumatoid arthritis, diabetes, and prostate cancer. Health economic aspects including costs, sick leave, and work performance have been studied, as have quality of life and socioeconomic aspects in relation to drug treatment. Important information on changes in the target population characteristics over time can be captured, and long-term trends in prescribing patterns can be followed and reliably linked to data from other sources. The importance of accurate background incidence estimates has also been analyzed (14).

Some QRs have published reports of clinical trials using randomization of patients within the QR, so-called randomized registry controlled trials (RRCTs), occasionally described as ‘a new disruptive scientific methodology’ (15,16). A growing awareness of methodological and data quality aspects in registry research has generated several publications taking national as well as multinational aspects into account.

## Discussion

The main finding of this survey is that Swedish National Quality of Care Registries may provide a feasible structure for managed introduction and long-term surveillance of new drugs and other medical interventions, including medical devices. The set-up, governance, and data management as well as in-registry clinical and scientific competence are of high quality as reflected by numerous publications in peer-reviewed journals. They can also meet a need for real-time clinical decision support. The registries are willing to collaborate with regulatory and health technology assessment (HTA) bodies by providing relevant data from clinical practice. However, so far only a limited number of QRs—e.g. those participating in this survey—have the capacity to deliver high-quality data at short notice, which might be important when answering important safety issues. Thus, conclusions from this study cannot automatically be extrapolated to all Swedish QRs. Further support is therefore needed to continue the improvement of registry quality and to expand the concept to additional therapeutic areas, which also will be in the interest of public health. To fully explore the potential of QR data, linkage to other sources of information is often needed. This sometimes adds further ethical and legal requirements, complicating study performances. By revising some of these regulations to comply with current medical and regulatory needs, registry data could be used more effectively.

There are similar registry set-ups in other European countries, and bi- or multinational collaborations are established or underway in several therapeutic areas. Results of such collaborations can be found in publications on MS (17), myocardial infarction (3), cancer risk in biologics-treated patients (18), and diabetes (19). Collaborations have also been extended to include methodological and study design issues (20). Taking these efforts into account, regulatory, HTA agencies, and other public institutions should consider supporting or even initiating multinational registry collaborations to answer specific questions, e.g. in orphan diseases or other situations with small study populations.

A particular strength of the Swedish QRs is the possibility to link data on individual patient characteristics with treatments and outcomes, including PROMs for several drugs and not just a single product, as is the case with product registries. Several registries directly involve patients in the development of PROMs, internet-based patient reporting, educational efforts, etc. (21). These common patients/registries initiatives could support the ongoing efforts by regulatory agencies, IMI projects, and other activities to involve patients in regulatory procedures further. The most obvious weakness from a regulatory perspective is the insufficient granularity of information on medications, in particular regarding dosing, formulations, and duration of treatment. However, data retrieved by linkage to the Prescribed Drug Registry can often compensate for this lack.

Methods for quality control need to be further harmonized between registries. One way to facilitate this could be by offering inspections of registries in line with GCP standards, another to facilitate studies to validate registry content. This could ultimately result in a certification as a ‘Good registry practice (GRP) registry’. A dialogue between regulators and registries may also facilitate the implementation of new EU regulations, for example the concept of ‘low interventional studies’ of obvious relevance to collection of clinical practice data in registries (22).

## Conclusions

Swedish health care quality registries contain useful information on drugs in clinical practice. This can be used to improve assessments made by regulatory agencies but also to support health policy and public health decision-making regarding drug-related issues. We propose that regulators should interact directly with representatives from the registries to elaborate their role in a regulatory context and discuss common efforts to improve quality and usefulness of registry data. Such a dialogue could stimulate a fruitful development where registries could contribute substantially to the evaluation of drug safety and effectiveness. Reliable post-marketing data collection is imperative for a life-cycle benefit–risk assessment of drugs and also to support managed introduction of new drugs in routine clinical care.

## Supplementary Material

Supplemental data
