# Supplementary material for: Utility of registries for post-marketing evaluation of medicines. A survey of Swedish health care quality registries from a regulatory perspective
Source: Ups J Med Sci. 2017 Mar 3;122(2):136–47. doi: 10.1080/03009734.2017.1285837 (PMC5441374; doi:10.1080/03009734.2017.1285837)
Supplement: Supplemental data [file IUPS_A_1285837_SM2463.zip › IUPS #1285837/IUPS _1285837 Feltelius Suppl MPA Quality Registry Questionnaire.pdf]

Survey[1] on SE quality registries drug information 2015

**1. Administrative information**		
**1. 1 Name on Quality registry in Swedish**		
**1.2 Name on Quality registry in English**		
**1. 3 Registry holder**		
**1. 4 Reporter**		
**Contact point/e-mail**		
**Reporting date**		
**1. 5 Scientific contact (if other than stated above)**		
**1. 6 Official website in Swedish**		
**1. 7 Is information concerning the registry available in English?**	Yes ☐	No ☐
If yes, please provide link to appropriate website		

**2. Basic information regarding current status of registry**
**2. 1** Which are the main diagnosis/indications included in the registry?

[1] Det går att förflytta sig genom dokumentet med följande tangenter: Tab., Page-up eller ”pil”. Enklast markeras kryssrutor med mellanslagstangenten. Dokumentet är låst för endast kunna fylla i befintliga ”gråmarkerade” textrutor, Word tappar då en del funktioner. Större textpartier skrivs lämpligen i annat dokument och klistras in här, alternativt kan hänvisning anges i resp. fråga till bifogad bilaga/appendix.

**2. 2** If your registry contain sub registries, please provide name on registry and main diagnosis/indication included:
**2. 3** Total cumulative no of patients included:
**2. 4** No of patients included per year (approx. mean last 3 years):
**2. 5** No of participating center's/clinics out of eligible units :
**2. 6** No of eligible center's/clinics?
**2. 7** Proportion of eligible patients (=coverage) included in the registry (%)
**2. 8** How is coverage calculated?

**3. Data content/elements:**	**Yes**	**No**	**Explanatory details/ Specification**
**3. 1** Are patients participating in ongoing RCTs included in the registry? (Y/N)<br>RCT= Randomized Clinical Trials	☐	☐	
**3. 2** Are RRCT possible to perform within your registry? (Y/N)<br>RRCT= Randomized Registry Control Trials	☐	☐	
**3. 3** Is it possible to create a control group? (Y/N)	☐	☐	
**3. 4** Are patients from other countries included in the registry? (Y/N)	☐	☐	
**3. 5** Demographic limitations e.g. age group, geographical? (Y/N)	☐	☐	
If Yes, please specify			

**4. Data elements registered at inclusion in registry**[2]	**Yes**	**No**	**Comments**
**4. 1** Age (Y/N)	☐	☐	
**4. 2** BMI (Y/N)	☐	☐	
**4. 3** Sex (Y/N)	☐	☐	

[2] If Yes, please specify measurements or if feasible refer to appendix/website

**4. 4** Diagnosis (Y/N)	☐	☐	
**4. 5** Comorbidities[2] (Y/N)	☐	☐	
**4. 6** What terminology for coding of diagnosis and comorbidity used is used?	☐ ICD-10<br>☐ Other<br>☐ Free text		
**4. 7** Time point for disease onset (Y/N)	☐	☐	
**4. 8** Disease activity/state[2] (Y/N)	☐	☐	
If Yes , please specify used measurement[2]			

**5. Medication - Drug of interest**	**Yes**	**No**	**Comments**
**5. 1** Is indication for treatment with drug of interest recorded in the registry? (Y/N)	☐	☐	
**5. 2** If yes, what terminology for coding of indication is used?			
**5. 3** What elements concerning medication are recorded?			
– Product (Y/N)	☐	☐	
– Substance (Y/N)	☐	☐	
– ATC code (Y/N)	☐	☐	
– Dosage (Y/N)	☐	☐	
– Duration/exposure (Y/N)	☐	☐	
– Therapy start/stop date (Y/N)	☐	☐	
– Is reason for stop/switch to other drug registered (Y/N)	☐	☐	
**5. 4** Is concomitant medication recorded in your registry? (Y/N)	☐	☐	

6. Elements registered at follow-up visits:	Yes	No	Comments
**6. 1** Is follow-up visits recorded in your registry? (Y/N)	☐	☐	
**6. 2** Are follow-up visits scheduled at regular intervals?	☐	☐	
If Yes, please specify intervals	Regularly ☐ If so, years or months<br>AD hoc ☐<br>Both ☐		
**6. 3** Are patients lost to follow-up registered? (Y/N)	☐	☐	
**6. 4** Are reasons for loss to follow-up registered? (Y/N)	☐	☐	
**6. 5** Maximum duration of long time follow-up?			
**6. 6** Is ongoing medication with drug of interest registered at follow-up? (Y/N)	☐	☐	
**6. 7** Is it possible to follow-up teratogenic events, due to medication with drug of interest? (Y/N)	☐	☐	
**6. 8** Is bio banking of DNA or tissue samples performed at inclusion (Y/N)	☐	☐	
**6. 9** Is bio banking of DNA or tissue samples performed at follow-up (Y/N	☐	☐	

7. Outcome measures[3]	Yes	No	*Specified measurements, for further details refer to appendix, if feasible*
**7. 1** Disease activity/state (Y/N)	☐	☐	
**7. 2** Organ damage e.g. renal damage (Y/N)	☐	☐	

[3] If Yes, please specify measurements or if feasible refer to appendix/website

**7. 3** Physical function (Y/N)	☐	☐	
**7. 4** Health economy/Cost-effectiveness data (Y/N)	☐	☐	
**7. 5** PROM (Y/N)<br>PROM= Patient Reported Outcome Measures	☐	☐	
**7. 6** PREM (Y/N)<br>PREM= Patient Reported Experiences Measures	☐	☐	
**7. 7** Is information on deaths and cause of death recorded? (Y/N)	☐	☐	
**7. 8** Quality of life (Y/N)	☐	☐	
**7. 9** Can you provide a summary list of used outcome measures in English, if needed (Y/N)	☐	☐	
**7. 10** Are the outcome measures the same as those used in clinical trials? (Y/N)	☐	☐	
**7. 11** Can long term safety be followed within your registry? (Y/N)	☐	☐	
**7. 12** If yes, is linkage to other data sources required? (Y/N)	☐	☐	

**8. Adverse event detection, processing and reporting**	**Yes**	**No**	**Comments**
**8. 1** Are adverse drug reactions (ADRs) registered within registry? (Y/N)	☐	☐	

If Yes – • Is an approved terminology for coding of ADRs used, i.e. MedDRA[4]? (Y/N)	☐	☐	
• Does the registry provides means for web-based reporting of ADRs directly to MPA[5]	☐	☐	
**8. 2** Are events (not ADRs) interfering with medication (surgery, accidents etc.) registered? (Y/N)	☐	☐	

9. Data capture/entry into database by:	Yes	No	Comments
**9. 1** Web-based reporting into database? (Y/N)	☐	☐	
**9. 2** Is it possible for patients to enter PROMs directly into the registry by the web? (Y/N)	☐	☐	
**9. 3** Is data in the registry extracted directly from Electronic health records? (Y/N)	☐	☐	
**9. 4** If yes, percentage of participating healthcare center's with this opportunity?			
**9. 5** Is the database an integrated part of an electronic patient record system? (Y/N)	☐	☐	

10. Quality control	Yes	No	Comments
**10. 1** Is there a specifically qualified person for quality control of data? (Y/N)	☐	☐	
If yes – please specify qualification (MD, research nurse, inspector etc.)			

[4] MedDRA= Medical Dictionary for Regulatory Activities *www.meddra.org/*
[5] MPA= Läkemedelsverket

**10. 2** Do you apply recommendations from specific international quality guidelines? (Y/N)	☐	☐	
If yes – please specify			
**10. 3** At what frequency is quality check performed (of raw data, delivery etc.)?	☐ Regularly, e.g. every six months<br>☐ Randomly<br>☐ Event driven		
**10. 4** At what level is registry data stored?<br>– Local/regional<br>– national<br>– multi-national	☐<br>☐<br>☐		
**10. 5** Is missing data actively requested? (Y/N)	☐	☐	
**10. 6** Do you consider it possible to request additional information from treating physician, if needed by external stake-holders (e.g. pharma companies)? (Y/N)	☐	☐	

**11. Data analyses**	**Yes**	**No**	**Comments**
**11. 1** Performed by contract research organisation? (Y/N)	☐	☐	
**11. 2** At what level is data pooled for analyses?	☐ Regional<br>☐ National<br>☐ Multi-national		

**12. Ethics**	**Yes**	**No**	**Comments**
**12. 1** Is there written patient <u>*information*</u>?	☐	☐	
**12. 2** Is a formal patient <u>*consent*</u> obtained?	☐	☐	

If yes, how<br>– Written consent<br>– Verbal consent	☐<br>☐	☐<br>☐	
**12. 3** Does consent include an agreement to ask for follow-up information by e.g. a questionnaire, when needed from stake-holders (e.g. pharma companies)? (Y/N)	☐	☐	
**12. 4** Has an ethics committee approved the working procedures/ protocols of your registry? (Y/N)	☐	☐	
**12. 5** Have your registry adopted any specific code of conduct e.g. Helsinki declaration or ENCEPPs code of conduct? (Y/N)	☐	☐	

**13. Legal and organizational aspects**	**Yes**	**No**	**Comments**
**13. 1** If available, please provide an organogram of your registry set-up			
**13. 2** Who owns the registry data?	☐ Health care professionals organisation<br>☐ Academic institution<br>☐ Pharma company<br>☐ Other		
**13. 3** Is there a formal (written) agreement between participating centers regulating data handling and analytic procedures? (Y/N)	☐	☐	
**13. 4** Do you collaborate with pharma companies, based on data from the registry? (Y/N)	☐	☐	
**13. 5** If yes, are the results used by companies for regulatory purposes? (Y/N)	☐	☐	
**13. 6** Is patient privacy protected by specific measures? (Y / N)	☐	☐	

**13. 7** If yes, how? Data/sample coding?			
**13. 8** Do you have a direct communication/ exchange of information with national regulatory agency (MPA)? (Y/N)	☐	☐	

**14. Communication and risk management**	**Yes**	**No**	**Comments**
**14. 1** Do you communicate results from your registry by scientific publications? (Y/N)	☐	☐	
**14. 2** If by other means, please specify e.g. annual report			
**14. 3** How many scientific publications has been published the last two years, based on data from your registry?			
**14. 4** When is feed- back given to reporting physician/clinics?			
– In real time (Y/N)	☐	☐	
– annually (Y/N)	☐	☐	
– AD HOC, in case of need e.g. safety problems (Y/N)	☐	☐	

**15. Financing by:**			**Approx. proportions (%) of total sum from each contributing part?**
**15. 1** Governmental/health care authorities? (Y/N)	☐	☐	
**15. 2** Industry? (Y/N)	☐	☐	
**15. 3** Research grants? (Y/N)	☐	☐	
