# Supplementary material for: Utility of registries for post-marketing evaluation of medicines. A survey of Swedish health care quality registries from a regulatory perspective
Source: Ups J Med Sci. 2017 Mar 3;122(2):136–47. doi: 10.1080/03009734.2017.1285837 (PMC5441374; doi:10.1080/03009734.2017.1285837)
Supplement: Supplemental data [file IUPS_A_1285837_SM2463.zip › IUPS #1285837/IUPS _1285837 Feltelius Suppl Table_Publications of regulatory significance.docx]

*Supplementary material for Feltelius N. et al. Utility of registries for post-marketing evaluation of medicines. A survey of Swedish health care quality registries from a regulatory perspective, Upsala Journal of Medical Sciences, 2017*

| **Table. List of publications of potential regulatory significance from registries in the survey, arranged by area of interest** | | |
| --- | --- | --- |
| ***Safety*** | | |
|  | | A Swedish national post-marketing surveillance study of natalizumab treatment in multiple sclerosis.  Holmen C, et al. A Mult Scler. 2011;17(6):708-19. |
|  | | 5-alpha reductase inhibitors, benign prostatic hyperplasia, and risk of male breast cancer.  Robinson D. et al. *Cancer causes & control. 2015* |
|  | | Insulin pump therapy, multiple daily injections, and cardiovascular mortality in 18,168 people with type 1 diabetes: observational study. Steineck I, et al. *BMJ*. 2015;350:h3234. |
|  | | Rheumatoid arthritis, anti-tumour necrosis factor therapy, and risk of malignant melanoma: nationwide population based prospective cohort study from Sweden. Raaschou P, et al. *BMJ*. 2013 |
|  | | Rheumatoid arthritis, anti-tumour necrosis factor treatment, and risk of squamous cell and basal cell skin cancer: cohort study based on nationwide prospectively recorded data from Sweden. Raaschou P, et al. *BMJ*. 2015 |
|  | | Warfarin-associated intracerebral hemorrhage after ischemic stroke. Åsberg S, et al. *Stroke.* 2014 |
|  | | Initial lymphocyte count and low BMI may affect fingolimod-induced lymphopenia.  Warnke C, et al. *Neurology*. 2014;83(23):2153-7 |
|  | | No association between rheumatoid arthritis, amyotrophic lateral sclerosis, and tumour necrosis factor inhibitor treatment. Arkema EV, et al. *Ann Rheum Dis*. 2014;73(11):2061-2. |
|  | | Risk and timing of cardiovascular disease after androgen-deprivation therapy in men with prostate cancer.  O'Farrell S, et al. *J Clin Oncol.* 2015;33(11):1243-51 |
| ***Effectiveness*** | | |
|  | | Durability of oral hypoglycemic agents in drug naïve patients with type 2 diabetes: report from the Swedish National Diabetes Register (NDR). Ekström N, et al. *BMJ Open Diabetes Res Care*. 2015 |
|  | | Treatment patterns and outcomes in patients undergoing percutaneous coronary intervention treated with prasugrel or clopidogrel (from the Swedish Coronary Angiography and Angioplasty Registry [SCAAR]).  Damman P, et al. *Am J Cardio*l. 2014;113(1):64-9. |
|  | | Persistent use of secondary preventive drugs declines rapidly during the first 2 years after stroke.  Glader EL,et al. *Stroke*. 2010;41(2):397-401. |
|  | | Antithrombotic Treatment Following Intracerebral Hemorrhage in Patients With and Without Atrial Fibrillation.  Pennlert J, et al. Stroke. 2015;46(8):2094-9 |
|  | | Comparative analysis of first-year fingolimod and natalizumab drug discontinuation among Swedish patients with multiple sclerosis. Frisell T, et al. *Multiple sclerosis.* 2016 |
|  | | Unfractionated heparin administration in patients treated with bivalirudin during primary percutaneous coronary intervention is associated lower mortality and target lesion thrombosis: a report from SCAAR.  Koutouzis M, et al. *Heart.* 2011 |
|  | | Effectiveness of TNF inhibitor switch in RA: results from the national Swedish register.  Chatzidionysiou K, et al. *Ann Rheum Dis*. 2014 |
|  | | Glycemic Control in Type 1 Diabetes and Long-Term Risk of Cardiovascular Events or Death After Coronary Artery Bypass Grafting. Nystrom T, et al. *J Am Coll Cardiol.* 2015;66(5):535-43 |
| ***Health economics*** | | |
|  | | How large are the productivity losses in contemporary patients with RA, and how soon in relation to diagnosis do they develop? Neovius M, et al, *Ann Rheum Dis.* 2011 |
|  | | Immediately versus delayed sequential bilateral cataract surgery. An analysis of costs and patient value.  Lundström M, et al. Acta Ophthalmol 2009 |
|  | | Cost-effectiveness of infliximab versus conventional combination treatment in methotrexate-refractory early rheumatoid arthritis: 2-year results of the register-enriched randomised controlled SWEFOT trial. Eriksson JK, et al *Ann Rheum Dis*. 2014 |
|  | | Impact of Socioeconomic Status on Cardiovascular Disease and Mortality in 24,947 Individuals With Type 1 Diabetes. Rawshani A, et al. *Diabetes Care*. 2015 |
| ***Methodology*** | | |
|  | | Incidence of rheumatoid arthritis in Sweden: a nationwide population-based assessment of incidence, its determinants, and treatment penetration. Eriksson JK, et al. *Arthritis Care Res (Hoboken)*. 2013 |
|  | | Ten years with biologics: to whom do data on effectiveness and safety apply? Simard JF, et al. *Rheumatology* (Oxford). 2011 |
|  | | The Swedish Web-system for enhancement and development of evidence-based care in heart disease evaluated according to recommended therapies (SWEDEHEART). Jernberg T, et al. *Heart*. 2010 |
|  | | Does the choice of EQ-5D tariff matter? A comparison of the Swedish EQ-5D-3L index score with UK, US, Germany and Denmark among type 2 diabetes patients. Kiadaliri AA, et al. *Health and Quality of Life Outcomes*. 2015 |
|  | | Risk of invasive melanoma in patients treated with biologics: results from a collaborative project of 11 European biologic registers. Mercer LK et al. Ann Rheum Dis 2016. |
|  | | Diabetes care--improvement through measurement. Eliasson B, et al. *Diabetes Res Clin Pract.* 2014 Dec;106 Suppl 2:S291-4. |
|  | | The National Diabetes Register in Sweden: an implementation of the St. Vincent Declaration for Quality Improvement in Diabetes Care. Gudbjörnsdottir S, et al. *Diabetes Care.* 2003 Apr;26(4):1270-6. |
|  |  | |
